# Parkinson’s disease–associated VPS35 mutant reduces mitochondrial membrane potential and impairs PINK1/Parkin-mediated mitophagy

**DOI:** 10.1186/s40035-021-00243-4

**Published:** 2021-06-15

**Authors:** Kai Yu Ma, Michiel R. Fokkens, Fulvio Reggiori, Muriel Mari, Dineke S. Verbeek

**Affiliations:** 1 Department of Genetics, University of Groningen, University Medical Center Groningen, Groningen, The Netherlands; 2 Department of Biomedical Sciences of Cells and Systems, University of Groningen, University Medical Center Groningen, Groningen, The Netherlands

**Keywords:** VPS35, PINK1, Parkin, Mitophagy, Mitochondrial membrane potential, Parkinson’s disease

## Abstract

**Background:**

Mitochondrial dysfunction plays a prominent role in the pathogenesis of Parkinson’s disease (PD), and several genes linked to familial PD, including *PINK1* (encoding PTEN-induced putative kinase 1 [PINK1]) and *PARK2* (encoding the E3 ubiquitin ligase Parkin), are directly involved in processes such as mitophagy that maintain mitochondrial health. The dominant p.D620N variant of vacuolar protein sorting 35 ortholog (VPS35) gene is also associated with familial PD but has not been functionally connected to *PINK1* and *PARK2*.

**Methods:**

To better mimic and study the patient situation, we used CRISPR-Cas9 to generate heterozygous human SH-SY5Y cells carrying the PD-associated D620N variant of *VPS35*. These cells were treated with a protonophore carbonyl cyanide m-chlorophenylhydrazone (CCCP) to induce the PINK1/Parkin-mediated mitophagy, which was assessed using biochemical and microscopy approaches.

**Results:**

Mitochondria in the VPS35-D620N cells exhibited reduced mitochondrial membrane potential and appeared to already be damaged at steady state. As a result, the mitochondria of these cells were desensitized to the CCCP-induced collapse in mitochondrial potential, as they displayed altered fragmentation and were unable to accumulate PINK1 at their surface upon this insult. Consequently, Parkin recruitment to the cell surface was inhibited and initiation of the PINK1/Parkin-dependent mitophagy was impaired.

**Conclusion:**

Our findings extend the pool of evidence that the p.D620N mutation of VPS35 causes mitochondrial dysfunction and suggest a converging pathogenic mechanism among VPS35, PINK1 and Parkin in PD.

**Supplementary Information:**

The online version contains supplementary material available at 10.1186/s40035-021-00243-4.

## Background

Parkinson’s disease (PD) is the second most common age-related neurodegenerative disorder, affecting more than 10 million people worldwide [1]. Most patients develop the disease in a sporadic manner through a complex interaction between genetic and environmental risk factors during ageing. Roughly 5%–10% of PD patients are caused by highly penetrant variants in genes such as *PINK1* (encoding PTEN-induced putative kinase 1 [PINK1]) and *PARK2* (encoding the E3 ubiquitin ligase Parkin) [2, 3]. This type of PD is referred to as familial PD, and missense variants of *VPS35* have been linked to the autosomal dominant form of familial PD [4, 5]. However, the c.1858G > A, p.D620N variant in *VPS35* is the only proven pathogenic variant [6]. *VPS35* encodes the vacuolar protein sorting-associated protein 35 (VPS35) that, together with VPS26 and VPS29, forms the cargo-selective subcomplex of the retromer complex [7]. The retromer recycles membrane proteins from endosomes to either the Golgi apparatus or the plasma membrane [8]. The p.D620N variant is located in a domain of VPS35 that is essential for protein–protein interactions [7]. Although the variant does not affect the formation of the retromer complex, it has impaired interactions with other factors such as the actin-nucleating WASH (Wiskott-Aldrich syndrome and SCAR homolog) complex [9, 10]. This leads to the altered retromer functioning and deficits in the sorting of cargoes [9–12].

Retromer also participates in the transport of mitochondrial cargoes to lysosomes or peroxisomes* via* mitochondrial-derived vesicles (MDVs) [13–15]. Previous reports have shown that VPS35 is involved in mitochondrial dynamics, as it recycles the fission protein DLP1 and regulates the level of the fusion protein MFN2 through the transport of mitochondrial E3 ubiquitin ligase 1 (MUL1) [14, 15]. Overexpression of the VPS35 D620N mutant augments mitochondrial fragmentation due to the increased DLP1 activity, whereas VPS35 depletion leads to mitochondrial fragmentation as a result of decreased level of MFN2, which correlates with a reduced mitochondrial respiratory capacity and a decrease in mitochondrial membrane potential [14–16].

Mitochondrial dysfunction plays an integral role in the pathogenesis of both sporadic and familial PD [17–19]. For example, loss-of-function variants of mitochondrial quality control genes such as *PINK1* and *PARK2* lead to early-onset autosomal recessive PD [2, 3, 20–22]. To maintain the mitochondrial quality, PINK1 is imported through a membrane potential–dependent process, from the outer mitochondrial membrane (OMM) into the inner mitochondrial membrane, where it is constitutively degraded by mitochondrial proteases [23, 24]. However, PINK1 import and cleavage is blocked upon mitochondrial depolarization caused by damage, resulting in the accumulation of PINK1 on the OMM. At the OMM, PINK1 phosphorylates ubiquitin and Parkin, leading to stable recruitment and activation of Parkin onto the mitochondrial surface [21, 24, 25]. Parkin then ubiquitinates different OMM substrates, inducing proteasomal degradation and removal of damaged cargoes *via* the MDVs-to-lysosome transport and/or mitophagy [26–28].

Mitophagy is a selective type of autophagy in which mitochondria targeted for degradation are sequestered into double-membrane autophagosomes and delivered into lysosomes [29, 30]. This process occurs in different physiological contexts [30]. For instance, most cells continuously undergo basal mitophagy during routine mitochondrial maintenance [31]. However, mitophagy can also be induced as a response to mitochondrial stressors such as mitochondrial depolarization. Notably, the PD-associated proteins PINK1 and Parkin are directly involved in stress-induced mitophagy [21, 24] but not in basal mitophagy [32, 33]. As dopaminergic neurons undergo substantial mitochondrial stress, presumably due to their pacemaker activity [34, 35], the stress-induced mitophagy *via* PINK1/Parkin has been heavily implicated in the pathogenesis of PD [30].

Given the mitochondrial impairments associated with the p.D620N variant of *VPS35* and the role of PINK1 and Parkin in maintaining mitochondrial quality control under stress conditions, we questioned whether the actions of these genes converge into a similar pathway to cause PD. Therefore, we set out to determine whether stress-induced mitophagy *via* PINK1/Parkin is affected by the VPS35 p.D620N mutant, using *VPS35* mutant SH-SY5Y cells carrying the p.D620N variant on one allele, which recapitulates the patient situation.

## Materials and methods

### Cell culture, transient transfections and treatments

Human SH-SY5Y neuroblastoma cells were maintained in Dulbecco’s Modified Eagle’s Medium (Invitrogen, Waltham, MA) supplemented with 15% fetal bovine serum (Invitrogen) and 1% Penicillin-Streptomycin (Gibco, Waltham, MA) in a 37 °C incubator with 5% CO_2_. Transient plasmid transfections were performed with plasmid DNAs using Lipofectamine (Thermo Fischer Scientific, Waltham, MA), according to the manufacturer’s instructions. To induce mitochondrial depolarization, the SH-SY5Y cells were treated with 10 μM or 20 μM carbonyl cyanide m-chlorophenylhydrazone (CCCP) (Sigma-Aldrich, Saint Louis, MO), 1 μM oligomycin (Sigma-Aldrich), 1 μM antimycin A (Sigma-Aldrich), or 1 μM antimycin A and 1 μM oligomycin (AO), for the indicated times, prior to cell harvesting or fixation. DMSO treatment was used as a control.

### Expression plasmids and antibodies

The plasmids used were pEGFP-Parkin [36] (a gift from Prof. Edward Fon (McGill University, Montreal, Quebec, Canada), Addgene plasmid #45875) and pEGFP-LC3 (a gift from Prof. Toren Finkel (University of Pittsburgh, Pittsburgh, PA), Addgene plasmid #24920) constructs [37]. The primary antibodies used for immunoblotting were mouse anti-ATPIF1 (1:1000, Abcam, Cambridge, UK, ab110277), mouse anti-β-actin (1:5000, MP Biomedicals, Irvine, CA, 8691001), mouse anti-β-tubulin (1:5000, Sigma-Aldrich T4026), mouse anti-Parkin (1:500, Santa-Cruz Biotechnology, Dallas, TX; sc-32,282), rabbit anti-PINK1 (1:1000, Cell signaling, Danvers, MA, #6946), mouse anti-TOM20 (1:500, BD Biosciences, San Jose, CA; 612278) and goat anti-VPS35 (1:1000, Abcam, ab10099). The primary antibodies used for immunofluorescence (IF) were mouse anti-TOM20 (1:200, Santa-Cruz Biotechnology sc-17764) and rabbit anti-PINK1 (1:200, Abcam ab216144). Secondary antibodies for immunoblotting were HRP-conjugated goat anti-rabbit IgG (H + L) (1:10000, Bio-Rad, Hercules, CA), HRP-conjugated goat anti-mouse IgG (H + L) (1:10000, Bio-Rad) and HRP-conjugated donkey anti-goat IgG (H + L) (1:10000, Abcam). Secondary antibodies for IF were Cy3-conjugated donkey anti-mouse IgG (H + L) (1:250, Jackson ImmunoResearch, West Grove, PA) and Alexa Fluor 488-conjugated donkey anti-rabbit IgG (H + L) (1:250, Jackson ImmunoResearch).

### Generation of VPS35 D620N/wild-type (WT) SH-SY5Y cells

The D620N mutation in the *VPS35* gene was obtained by Clustered Regularly Interspaced Short Palindromic Repeats (CRISPR)-Cas9–mediated genome editing in the SH-SY5Y neuroblastoma cell line, as previously described [38]. Briefly, a 20-nt single guide RNA (sgRNA) sequence that targets exon 15 of the *VPS35* gene and is predicted to cut approximately 9 base-pairs (bp) upstream of the GAT triplet encoding the aspartic acid residue on location 620 was cloned into the pSpCas9(BB)-2A-GFP (PX458) plasmid (a gift from Prof. Feng Zhang (Broad Institute, Cambridge, MA); Addgene plasmid #48138) using the *BbsI* restriction enzyme to form the targeting plasmid expressing Cas9-GFP. In addition, a single-stranded oligodeoxynucleotide sequence was designed to facilitate homology-directed repair of the endogenous locus and included the substitution of five nucleotides: a nucleotide substitution G > A that leads to the D620N mutation of *VPS35* and four synonymous substitutions that create a novel *EcoRI* restriction site that also destroys the protospacer-adjacent motif sequence to avoid repetitive cutting of Cas9 by the repair template. Following validation, the PX458-sgRNA plasmid and the single-stranded oligonucleotides were transfected into the SH-SY5Y cells following the manufacturer’s protocol (Lonza, Basel, Switzerland). GFP-positive cells were single-cell sorted 48 h post-transfection using a SH800S cell sorter (Sony Biotechnology, San Jose, CA) and grown in separate cultures that were subsequently screened for the D620N mutation using the restriction enzyme *EcoRI*. In parallel, we mock-electroporated and sorted the same batch of cells, which were used as WT control in the following experiments. Finally, we sequenced the top three predicted off-target genomic regions within coding regions (obtained from http://crispr.mit.edu) of genes *POU6F1, ZNF318* and *KY*, but found no off-target edits (not shown). Detailed primer and template sequences are provided in Table S1.

### Generation of stable COX8-EGFP-mCherry reporter SH-SY5Y cells

The COX8-EGFP-mCherry sequence was obtained from the pCLBW COX8-EGFP-mCherry construct [39] (a gift from Prof. David Chan (Caltech, Pasadena, CA), Addgene plasmid #78520) through restriction enzyme digestion with *ApaI* and *EcoRI*, and was ligated into the mammalian expression vector pcDNA 3.1(+). Subsequently, the vector was transfected into WT and VPS35^D620N^ SH-SY5Y cells using Lipofectamine 3000 (Thermo Fischer Scientific), following the manufacturer’s protocol. Forty-eight hours after transfection, the growth medium was replaced with selection medium containing 800 ng/μl G-418 (Sigma-Aldrich). The selection medium was refreshed every other day for 10 days until only cells with the plasmid remained. Stable cell lines were cultured for three passages before performing the experiments.

### Protein extraction and immunoblotting

SH-SY5Y cells were harvested in 2% sodium dodecyl sulfate (SDS)/phosphate-buffered saline (PBS) buffer containing a proteinase inhibitor cocktail (Roche, Basel, Switzerland) and sonicated. Crude mitochondrial fractions were isolated as previously described [40]. Briefly, SH-SY5Y cells were collected and homogenized using a Dounce homogenizer in ice-cold isolation buffer containing 320 mM sucrose and a proteinase inhibitor cocktail. The homogenized samples were differentially centrifuged at 1500 g for 15 min and 17,000 g for 30 min to obtain nuclei and crude mitochondria, respectively. The cytosolic fraction was obtained from the final supernatant. Protein concentrations were quantified using the Pierce™ BCA protein assay kit (Thermo Fischer Scientific), and samples were mixed with loading buffer containing 10% β-mercaptoethanol before being boiled at 95 °C for 5 min. Subsequently, equal amounts of total protein extracts were subjected to SDS-PAGE, transferred to nitrocellulose membranes, blocked for 1 h in skimmed milk, incubated overnight with primary antibody at 4 °C and then with the corresponding secondary antibody for 1 h at room temperature (RT). The blots were imaged on a Chemidoc™ MP Imaging System (Bio-Rad). Protein levels were quantified by densitometry using the ImageJ software (NIH, Bethesda, MD).

### Immunofluorescence

WT and VPS35^D620N^ SH-SY5Y cells that were seeded on glass coverslips in 24-well plates were fixed in 4% paraformaldehyde in PBS for 10 min at RT. Cells were then permeabilized in 0.1% Triton X-100 in PBS for 10 min and blocked with 5% donkey serum (Abcam) in PBS for 1 h. The coverslips were then incubated overnight at 4 °C with the primary antibodies diluted in blocking buffer and for 1 h at RT for secondary antibody incubation. Coverslips were finally mounted onto glass slides in 4′,6-diamidino-2-phenylindole (DAPI)-containing mounting medium (Vector Laboratories, Burlingame, CA). The slides were analyzed using either structured illumination microscopy (SIM) or confocal microscopy. SIM images were acquired with an AxioObserver Z1 compound microscope (Carl Zeiss, Oberkochen, Germany) equipped with an Apotome, 63x oil objective and an AxioCam MRm3 CCD camera (Carl Zeiss). Confocal images were acquired with a TCS SP8 high-resolution confocal laser scan microscope (Leica Microsystems, Wetzlar, Germany) and an HC PL APO CS2 63x/1.4 oil objective. For quantitative analysis, maximum intensity projections were generated from all Z-stacks, which were captured for each condition with identical exposure times or laser settings.

### Image analysis

All image analyses were performed using the ImageJ software (NIH). Colocalization analyses of PINK1 and TOM20 were performed using ImageJ plugin Coloc 2 (https://imagej.net/Coloc_2). Regions of interest (ROIs) were created per cell in the TOM20 channel (*n* = ~ 80–100 cells per condition in each experiment). Pearson’s correlation coefficients were subsequently determined per ROI using the Costes method for threshold regression [41].

Mitochondrial morphology was quantified as previously described [42]. Briefly, images of single cells were pre-processed and binarized, followed by particle analysis and computation of several metrics. The number of mitochondria was determined as the number of individual particles. The aspect ratio was determined by dividing the major axis by the minor axis of each particle. A total of 80–100 cells were quantified per condition in each experiment.

For the EGFP-Parkin translocation experiment, a blinded observer scored each cell for either diffuse EGFP-Parkin or mitochondria-localized EGFP-Parkin (*n* = ~ 50 cells per condition in each experiment), as previously described [36].

Mitophagy in COX8-EGFP-mCherry stable cell lines was quantified by determining the ratio of the number of particles obtained from the mCherry channel (mitophagolysosomes) to the number of particles obtained from the EGFP channel (mitochondria) per cell (*n* = ~ 50–70 cells per condition in each experiment). Particles were analyzed in a similar fashion to the mitochondrial morphology quantification.

EGFP-LC3 puncta on mitochondria were quantified as follows: a mask was created from the TOM20 mitochondrial staining and used as overlay over the EGFP-LC3 image. The puncta were subsequently counted for each cell (*n* = ~ 40–50 cells per experiment).

### Mitochondrial membrane potential quantification

Mitochondrial membrane potential was measured using fluorescence-activated cell sorting (FACS). WT and VPS35^D620N^ SH-SY5Y cells were incubated for 30 min with 100 nM tetramethylrhodamine methyl ester (TMRM) dye (Thermo Fischer Scientific) and 100 nM MitoTracker Green FM dye (Thermo Fischer Scientific) diluted in culture medium. Cells were rinsed, dissociated with 0.05% Trypsin-EDTA (Thermo Fischer Scientific) and aliquoted in multiple FACS tubes. FACS measurements were performed with a FACSCalibur flow cytometer (BD Biosciences) or a Novocyte Quanteon flow cytometer (Agilent, Santa Clara, CA ). For the timeline measurements, baseline measurements were taken, after which CCCP was added to a final concentration of 10 μM, upon which measurements were taken at each time point. For the dose-response measurements, CCCP was added to the indicated final concentrations, and 1 min later measurements were made. Slopes were determined using a simple linear regression method. For 24-h treatments, cells were treated with 10 μM CCCP or 1 μM antimycin A and 1 μM oligomycin for 24 h, then the cells were dissociated and TMRM was measured as indicated above. Three independent sorts measuring at least 10,000 cells were performed per clone per data point for all experiments. Data analysis was performed using the Kaluza Analysis software (Beckman Coulter, Brea, CA). Mitotracker Green median fluorescence intensity was used to correct for mitochondrial mass fluctuations.

### Ultrastructural analyses

For conventional transmission electron microscopy (TEM), WT and VPS35^D620N^ SH-SY5Y cells were treated with DMSO or 10 μM CCCP for 6 h. Then an equal volume as the culture media of double-strength fixative (4% paraformaldehyde, 5% glutaraldehyde in 0.1 M sodium cacodylate buffer, pH 7.4) was then added to the cells and incubated for 20 min at RT, followed by further fixing the cells with the same volume of single-strength fixative (2% paraformaldehyde, 2.5% glutaraldehyde in 0.1 M sodium cacodylate buffer, pH 7.4) for 2 h at RT. After five washes with 0.1 M sodium cacodylate buffer (pH 7.4), the cells were scraped and embedded as previously described [43]. Subsequently, 70-nm ultrathin sections were cut using a Leica EM UC7 ultra microtome (Leica Microsystems) and stained with uranyl acetate and lead citrate as previously described [43]. The cell sections were analyzed using an 80 kV transmission electron microscope CM100bio TEM (FEI, Eindhoven, The Netherlands).

The analysis of the different mitochondrial profiles per cell type was performed by random screening of sections derived from at least three different grids per sample. The mitochondrial profiles were categorized as follows: classical mitochondria with well-defined cristae (category I), dark mitochondria with well-defined cristae often swelling (category II), mitochondria with undefined cristae (category III), dark mitochondria with undefined contours and cristae (category IV), and large mitochondria with very light content and few remnant cristae (category V). The number of each mitochondrial type per condition was determined by counting 665, 579 and 521 mitochondria profiles from the DMSO-treated WT and VPS35^D620N^ cell (clones 1 and 2) sections, respectively, as well as 727, 1028 and 914 mitochondria profiles from the CCCP-treated WT and VPS35^D620N^ cell (clones 1 and 2) sections, respectively.

### Cell viability assay

Cell viability upon treatment with CCCP was determined using a 3-(4,5-demethylthiazol-2-yl)-2,5-diphenyltetrazolium bromide (MTT) reduction assay (Abcam). The SH-SY5Y cells were plated in 96-well plates 12 h prior to incubation with CCCP for 24 h. MTT assay compounds were added following the manufacturer’s protocol and absorbance was measured using a Synergy HT optical plate reader (Biotek, Winooski, VT).

### Statistical analyses

Data of Western blot densitometry measurements, mitochondrial membrane potential measurements, TEM and mitochondrial morphology were analyzed using a linear model by one-way or two-way analysis of variance (ANOVA) followed by Tukey’s *post-hoc* test. Count data, such as the EGFP-LC3 and mitochondrial particle quantification data were modelled using a generalized linear model followed by one-way or two-way ANOVA and Tukey’s *post-hoc* test. PINK1-TOM20 colocalization data were analyzed using the Kruskal-Wallis test followed by pairwise Mann-Whitney U-test with Benjamini-Hochberg multiple testing correction. Proportional data of COX8-EGFP-mCherry mitophagy and EGFP-Parkin localization were analyzed using beta regression analysis. Data are means ± SEM from at least three independent experiments, unless otherwise specified. *P* < 0.05 was considered as statistically significant. Statistical analyses were performed in the statistical computing environment R (version 1.3.959; The R Foundation for Statistical Computing; Vienna, Austria).

## Results

### Generation of heterozygous *VPS35* D620N SH-SY5Y cells

**Fig. 1:**
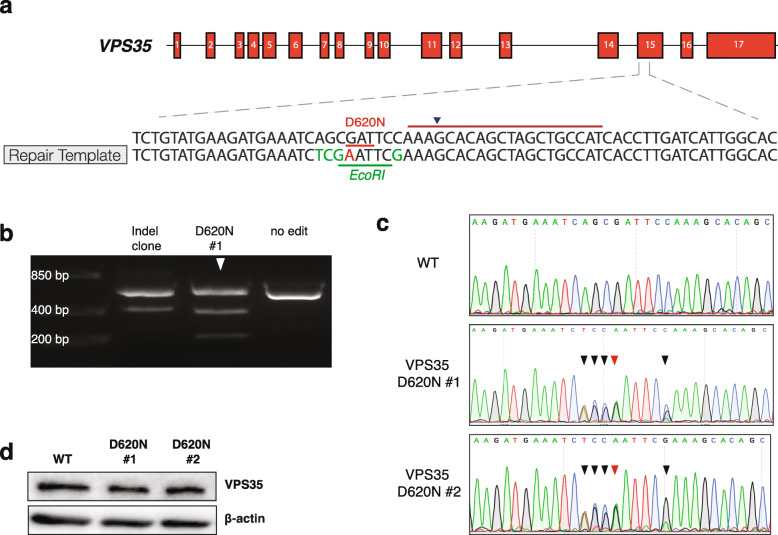
Generation of heterozygous VPS35^D620N^ SH-SY5Y cells. **a** Schematic representation of cloning strategy for the generation of nucleotide exchange in the *VPS35* locus that leads to the D620N mutation using CRISPR-Cas9 technology. Partial sequence of endogenous target on exon 15 is shown (upper sequence). The 20-nt guide sequence is depicted by the burgundy line with the blue arrowhead pointing towards the predicted Cas9-cleavage site. GAT sequence that encodes the aspartic acid residue at position 620 is highlighted in red. Lower sequence shows the design of the repair template used to create the mutation. The substitution G > A is shown in red. Silent substitutions to create an *EcoRI* restriction site are shown in green. **b** Representative agarose gel showing PCR products of the target *VPS35* region obtained from different CRISPR-clones. PCR products were subjected to *EcoRI* digestion before loading on an agarose gel. Upper bands show noncleaved bands of the PCR amplicon. Arrowhead indicates an example of a successfully edited clone (VPS35^D620N^ clone 1) that was partially cleaved by *EcoRI*. **c** Sanger chromatograms of the region surrounding the CRISPR-edit of WT (upper panel), VPS35 D620N clone 1 (middle panel) and VPS35 D620N clone 2 (lower panel). Red arrowhead indicates successful integration of the PD-associated mutation G > A. Black arrowheads indicate silent substitutions to create the *EcoRI* restriction site. Note that double peaks are shown for the edited nucleotides as one allele remains unaltered. **d** Representative immunoblots of protein extracts from WT and VPS35^D620N^ (clone 1 and clone 2) cells. Blots were stained for VPS35 and β-actin (total protein loading control)

To date, most studies have investigated the p.D620N variant in *VPS35* (VPS35^D620N^) by stably overexpressing VPS35^D620N^ in in vitro and in vivo models. However, the enhanced VPS35 levels in these models may affect the retromer functioning, as higher or lower levels of VPS35 correlate with alterations in mitochondrial fragmentation [14, 15]. This motivated us to use CRISPR-Cas9–mediated genome editing to introduce the p.D620N variant in *VPS35* into the human neuroblastoma SH-SY5Y cells widely used in PD research [38, 44] (Fig. 1a). Restriction fragment length polymorphism analysis using the *EcoRI* enzyme on a 604-bp genomic DNA region surrounding the variant revealed two putative positive clones (Fig. 1b). Sanger sequencing validated the presence of the p.D620N variant, resulting in a GAT-to-AAT codon change, in only one of two *VPS35* alleles, mimicking the heterozygous carrier status seen in patients (Fig. 1c). Additionally, immunoblotting showed that the introduction of the p.D620N variant did not affect the expression level of VPS35 compared to the WT cells (Fig. 1d). These cell lines were used for the experiments of this study.

### PINK1-mediated Parkin recruitment to mitochondria is impaired in the CCCP-treated VPS35^D620N^ cells

**Fig. 2:**
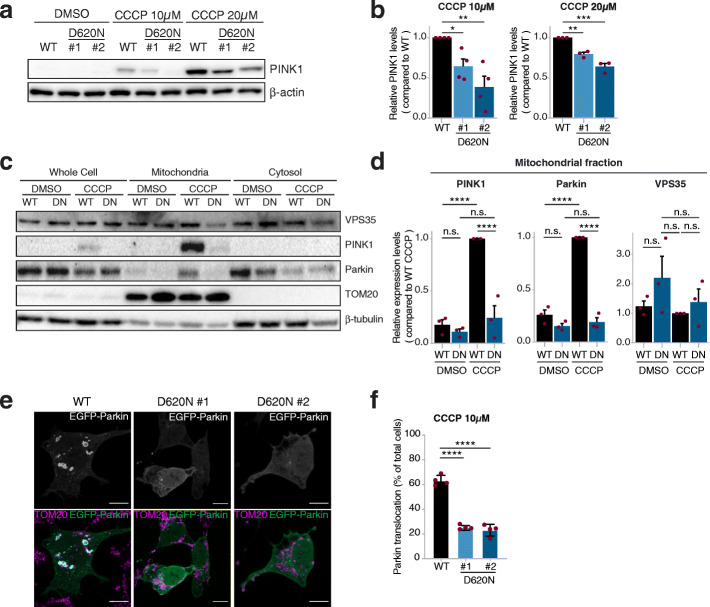
CCCP-induced PINK1/Parkin recruitment is impaired in VPS35^D620N^ cells. **a** Representative immunoblots for PINK1 in WT and VPS35^D620N^ (clone 1 and clone 2) cells treated with DMSO, 10 μM or 20 μM CCCP for 24 h. **b** Quantification of PINK1 levels from immunoblots in (**a**). Shown are relative PINK1 levels after 10 μM or 20 μM CCCP stimulation for 24 h compared to WT cells. Note that PINK1 levels were not quantifiable when treated with DMSO and thus comparisons were made within each treatment condition. Each red dot depicts a separate experiment. Statistical analyses were performed by one-way ANOVA followed by Tukey’s *post-hoc* test. **c** Representative immunoblots for VPS35, PINK1, Parkin, TOM20 (mitochondrial loading control) and β-actin (total protein loading control) using protein extracts derived from whole cell, crude mitochondrial fractions or cytosolic fraction from WT and VPS35^D620N^ cells treated with either DMSO or 10 μM CCCP for 6 h. **d** Quantification of PINK1, Parkin and VPS35 in the mitochondrial fraction from the immunoblots in (**c**). Shown are relative levels compared to the WT cells treated with CCCP. Each red dot depicts a separate experiment. Statistical analyses were performed by two-way ANOVA followed by Tukey’s *post-hoc* test. **e** Representative fluorescence images of cells transiently overexpressing EGFP-Parkin (upper panels: white, lower panels: green), which were also stained for the mitochondrial protein TOM20 (lower panels: magenta). WT cells with EGFP-Parkin translocated onto mitochondria are shown in the left panels and VPS35^D620N^ cells showing a diffuse cytosolic distribution of EGFP-Parkin are shown in the middle (clone 1) and right (clone 2) panels. Scale bar, 10 μm. **f** Quantification of WT and VPS35^D620N^ (clone 1 and clone 2) cells with EGFP-Parkin translocation to mitochondria, as shown in (**e**), after 10 μM CCCP stimulation for 24 h, represented as fraction (%) of all counted cells. Red dots represent the means of four different experiments in which at least 50 cells were counted. Data were analyzed using a beta regression. *P < 0.05, **P < 0.01, ***P < 0.005, ****P < 0.001

To investigate whether the p.D620N variant in VPS35 affects the PINK1/Parkin-mediated mitophagy, we used the protonophore CCCP to induce mitochondrial stress by dissipating the mitochondrial membrane potential (Δψ_m_) and thereby activate PINK1/Parkin-mediated mitophagy [21, 24, 25]. We used immunoblotting to investigate PINK1 accumulation over time in the WT and VPS35^D620N^ cells upon 10 μM CCCP treatment. As expected, the total PINK1 level increased slightly after 3 h of CCCP treatment, and PINK1 accumulation was pronounced after 24 h of CCCP treatment in the whole cell extracts and crude mitochondrial fractions of WT cells (Fig. 2c, d; Fig. S1a). This coincided with Parkin accumulation in the crude mitochondrial fraction (Fig. 2c, d) and a decrease in the whole lysate (Fig. S1b, c; Fig. 2c), likely due to the autoubiquitination and increased proteasomal turnover of mitochondria-bound Parkin [45]. However, the total PINK1 level was substantially lower in the whole extracts and crude mitochondrial fractions of CCCP-treated VPS35^D620N^ cells at both time points compared to the WT cells (Fig. S1a, Fig. 2a–d). Likewise, the total Parkin levels remained similar to those in the untreated condition (Fig. S1a, Fig. 2c). Of note, the VPS35 levels did not change upon CCCP treatment (Fig. S1a; Fig. 2c, d), and VPS35 was present in the crude mitochondrial fraction (Fig. 2c), consistent with previous reports [14, 15].

Previous studies have shown a dose-dependent effect of CCCP and thus we questioned whether a higher dose of CCCP would be able to stabilize PINK1 on mitochondria in the VPS35^D620N^ cells. Indeed, 20 μM CCCP led to higher PINK1 levels compared to 10 μM CCCP in WT cells after 24 h of treatment and marked PINK1 accumulation in the VPS35^D620N^ cells (Fig. 2a, b). However, the total PINK1 level in the VPS35^D620N^ cells remained significantly lower than those in the WT cells (Fig. 2a, b). Consistent with the increased PINK1 level upon 20 μM CCCP treatment, the proteasomal degradation of Parkin in the WT cells also further increased with 20 μM CCCP [45], which was not observed in the VPS35^D620N^ cells (Fig. S1b, c). Of note, 20 μM CCCP demonstrated increased cytotoxicity compared to 10 μM CCCP (Fig. S1d). These data suggest that 10 μM CCCP induces milder damage to mitochondria than 20 μM CCCP and exposes a not-yet-characterized deficit in the VPS35^D620N^ clones.

**Fig. 3:**
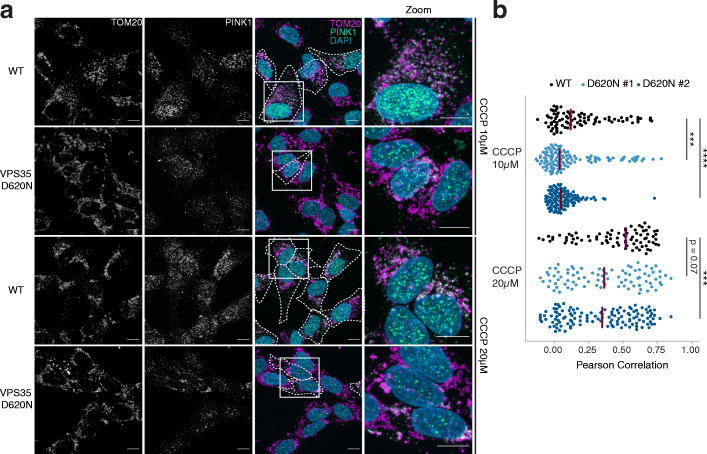
PINK1 does not localize to mitochondria upon CCCP treatment in VPS35^D620N^ cells. **a** Representative fluorescence images of WT or VPS35^D620N^ (clone 1) cells treated with 10 μM or 20 μM CCCP for 24 h. From left to right: endogenous TOM20 staining; endogenous PINK1 staining; overlay image including DAPI staining for nuclei in which cells with co-localization (white) of TOM20 (magenta) and PINK1 (green) are outlined; zoom-in of highlighted area in overlay image. Scale bar, 10 μm, or 4 μm for zoomed images. **b** Quantification of co-localization of endogenous TOM20 and PINK1 of WT and VPS35^D620N^ (clones 1 and 2) cells treated with 10 μM or 20 μM CCCP for 24 h. Each dot represents the Pearson coefficient calculated for one cell. Red lines show the median Pearson coefficient per cell line. Statistical analyses were performed using the Kruskal-Wallis test followed by a pairwise Mann-Whitney U-test with Benjamini-Hochberg multiple testing correction. ***P < 0.005, ****P < 0.001

Next, we used IF to quantify the translocation of cytosolic EGFP-Parkin to mitochondria upon treatment with CCCP, since endogenous Parkin was not detectable in our cells. WT and VPS35^D620N^ cells were transiently transfected with EGFP-Parkin and subsequently treated with 10 μM CCCP for 6 h and stained for OMM protein TOM20. As expected, mitochondrial depolarization due to CCCP caused translocation of cytosolic EGFP-Parkin to mitochondria in WT cells, as shown by the colocalization between EGFP-Parkin and TOM20 (Fig. 2e). Additionally, less Parkin translocation was seen in the VPS35^D620N^ cells (± 23% and ± 25%) compared to the WT cells (±63%) (Fig. 2f). We further examined colocalization between endogenous PINK1 and TOM20 using IF in WT and VPS35^D620N^ cells upon CCCP treatment (Fig. 3a). The CCCP-treated VPS35^D620N^ cells showed less colocalization between PINK1 and TOM20 compared to the WT cells, with a dose-dependent effect of CCCP (Pearson correlation coefficient: 10 μM CCCP, WT median 0.12 vs clone 1 median 0.04 and clone 2 0.05; 20 μM CCCP, 0.52 vs 0.36 and 0.35) (Fig. 3b). Together, these data suggest that the CCCP-induced PINK1 accumulation is hampered in VPS35^D620N^ cells, leading to impaired Parkin recruitment onto the mitochondria.

### CCCP-induced mitophagy is impaired in VPS35^D620N^ cells

**Fig. 4:**
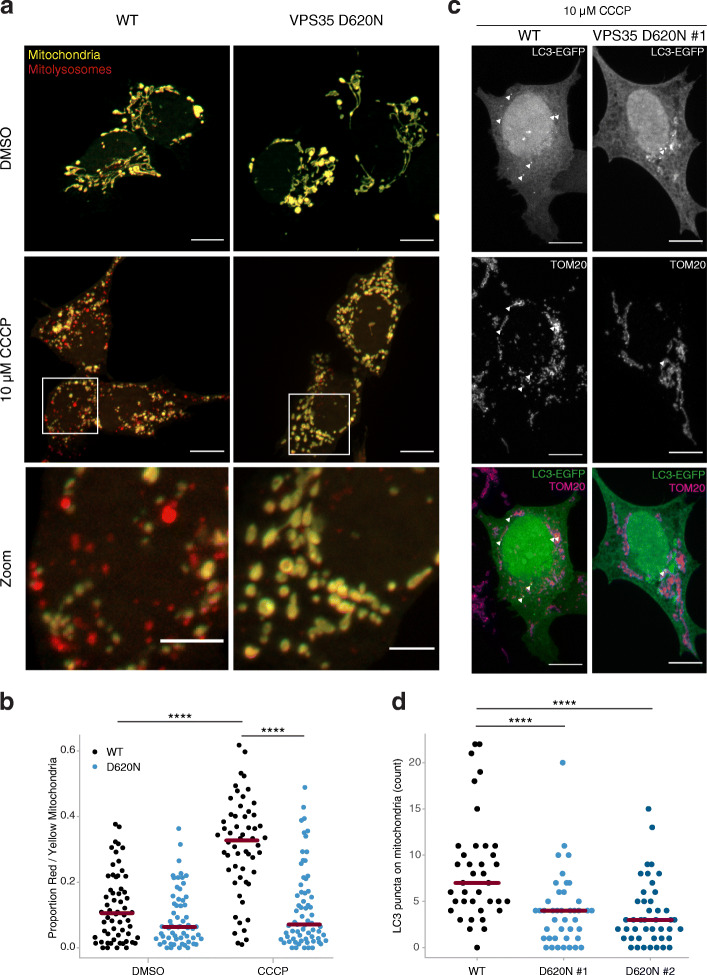
VPS35^D620N^ cells display impaired mitophagy upon CCCP treatment. **a** Representative fluorescence images of WT (left panels) or VPS35^D620N^ (right panels) cells stably expressing COX8-EGFP-mCherry treated with DMSO (upper panels) or 10 μM CCCP (middle panels) for 24 h. The signal from the yellow particles originates from EGFP and mCherry and highlights cytoplasmic mitochondria. The red particles show quenched EGFP signal and normal mCherry signal, reflecting mitochondria transported into an acidic compartment (mitophagolysosome). Lower panels show zoomed-in images from the highlighted area in the middle panels. Scale bar, 10 μm, or 4 μm for zoomed images. **b** Quantification of yellow and red particles in (**a**) in WT and VPS35^D620N^ cells treated with DMSO or 10 μM CCCP. Each dot represents the proportion of total yellow and red particles for one cell. Red lines show the median proportion per cell line per condition. Statistical analysis was performed using a beta regression. **c** Representative confocal images of WT (left panels) or VPS35^D620N^ (clone 1, right panels) cells transiently overexpressing EGFP-LC3 (upper panel: white, lower panel: green) and stained for the mitochondrial protein TOM20 (middle panel: white, lower panel: magenta) treated with 10 μM CCCP for 6 h. Scale bar, 10 μm. **d** Quantification of EGFP-LC3 puncta colocalized with mitochondria in WT and VPS35^D620N^ (clone 1 and 2) cells treated with 10 μM CCCP. Each dot represents one cell. Red lines show the median amount of EGFP-LC3 on mitochondria per cell line. Data analyzed using one-way ANOVA. ****P < 0.001

To prove that the hampered PINK1 and Parkin recruitment onto mitochondria upon CCCP treatment does lead to compromised PINK1/Parkin-mediated mitophagy in VPS35^D620N^ cells, we used previously published dual color fluorescence-quenching EGFP-mCherry mitophagy reporter [39], and stably expressed it in the WT and VPS35^D620N^ cells. Under normal conditions, mitochondria emitted both a red and a green fluorescence signals, resulting in a yellow color (Fig. 4a). Mitochondria damaged by CCCP treatment were transported to lysosomes for degradation, and the EGFP fluorescent signal was quenched within this acidic organelle, leaving mainly a red fluorescent signal (Fig. 4a, b). At steady state, both WT and VPS35^D620N^ cells primarily showed a yellow reticulated mitochondrial network, with only a few red-only puncta, probably reflecting mitochondria within lysosomes, i.e. mitolysosomes, and there was no significant difference between the cell lines (Fig. 4a, b). In contrast, after 24 h of 10 μM CCCP treatment, WT cells showed a substantial increase in mitochondria with a red-only signal, indicating activation of mitophagy [39], while VPS35^D620N^ cells did not display a shift from yellow to red-only mitochondria (Fig. 4a, b). Interestingly, punctate rearrangement of the mitochondrial network was observed in the VPS35^D620N^ cells after CCCP treatment, in which the mitochondrial clumps seemed larger compared to the WT cells (Fig. 4a, right bottom panel compared to left bottom panel). This suggests that VPS35^D620N^ cells do react to CCCP but experience impairment in PINK1/Parkin-mediated mitophagy.

To confirm this finding, we investigated mitophagy using a different approach by transiently transfecting WT and VPS35^D620N^ cells with EGFP-LC3, a protein marker for autophagosomes [46]. Mitophagy was induced by 10 μM CCCP treatment for 6 h, and we subsequently used IF to examine the colocalization between LC3 puncta and TOM20 (Fig. 4c, arrowheads). CCCP treatment in the WT cells led to approximately twice the amount of LC3- and TOM20-positive mitophagosomes compared to the VPS35^D620N^ cells (Fig. 4d). Moreover, multiple VPS35^D620N^ cells did not form TOM20-positive autophagosomes, a phenomenon rarely seen in the WT cells (Fig. 4d). Altogether, these results confirm that the CCCP-induced mitophagy is impaired in VPS35^D620N^ cells.

### VPS35^D620N^ cells accumulate PINK1 in response to mitochondrial depolarization caused by antimycin A and oligomycin

**Fig. 5:**
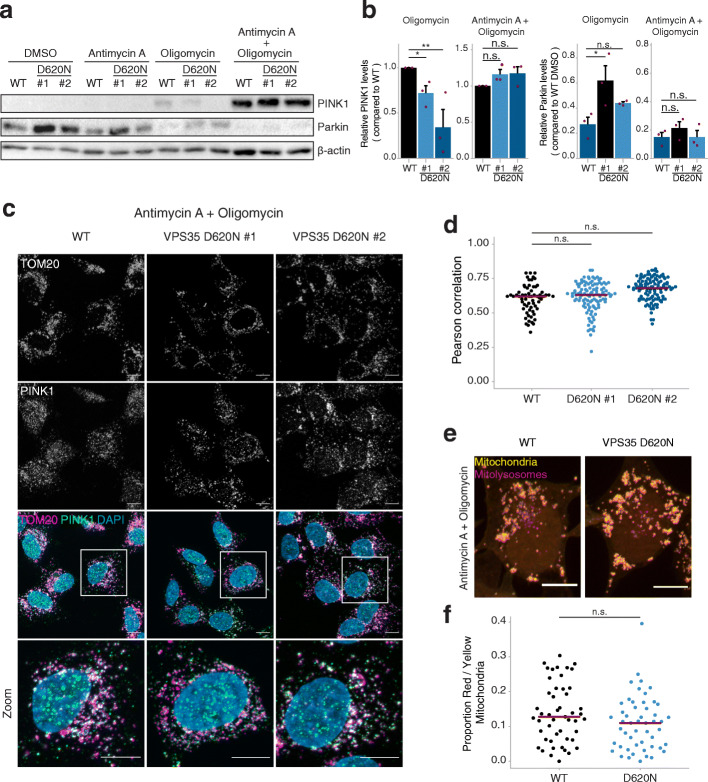
PINK1 accumulation is unaltered in VPS35^D620N^ cells as a result of depolarization *via* antimycin A and oligomycin. **a** Representative immunoblots of protein extracts from WT and VPS35^D620N^ (clone 1 and clone 2) cells treated with DMSO, 1 μM antimycin A, 1 μM oligomycin, or a combination of both drugs for 24 h. Blots were stained with PINK1, Parkin and β-actin (total protein loading control) antibodies. **b** Quantification of PINK1 and Parkin levels from immunoblot analysis in (**a**). Shown are relative PINK1 and Parkin levels after 1 μM oligomycin or 1 μM antimycin A and 1 μm oligomycin stimulation for 24 h compared to the WT cells. Each red dot depicts a separate experiment. Statistical analyses were performed by one-way ANOVA followed by Tukey’s *post-hoc* test. **c** Representative fluorescence images of WT or VPS35^D620N^ (clone 1 and 2) cells treated with 1 μM antimycin A and 1 μM oligomycin. From top to bottom: endogenous TOM20 staining; endogenous PINK1 staining; overlay image of TOM20 (magenta), PINK1 (green) and DAPI staining for nuclei (blue); zoom in of highlighted area in overlay image. Scale bar, 10 μm, or 4 μm for zoomed images. **d** Quantification of co-localization of endogenous TOM20 and PINK1 in WT and VPS35^D620N^ (clones 1 and 2) cells treated with 1 μM antimycin A and 1 μM oligomycin, as shown in (**c**). Each dot represents the Pearson coefficient calculated for one cell. Red lines show the median Pearson coefficient per cell line. Statistical analysis was performed using a Kruskal-Wallis test followed by a pairwise Mann-Whitney U-test with Benjamini-Hochberg multiple testing correction. **e** Representative fluorescence images of WT (left panels) or VPS35^D620N^ (right panels) cells that stably express COX8-EGFP-mCherry treated with 1 μM antimycin A and 1 μM oligomycin for 24 h. The signal from the yellow particles originates from EGFP and mCherry and highlights cytoplasmic mitochondria. The red particles show quenched EGFP signal and normal mCherry signal, reflecting mitochondria transported into an acidic compartment (mitophagolysosome). Scale bar, 10 μm. **f** Quantification of yellow and red particles in WT and VPS35 D620N cells in (**e**). Each dot represents the proportion of total yellow and red particles for one cell. Red lines show the median proportion per cell line per condition. Statistical analysis was performed using a beta regression. n.s. non-significant, *P < 0.05, **P < 0.01

Next, we questioned if PINK1/Parkin-mediated mitophagy in VPS35^D620N^ cells would be impaired by treatment with two agents that, like CCCP, also lead to substantial mitochondrial depolarization: subcomplex III inhibitor antimycin A and F_1_F_0_ ATPase inhibitor oligomycin [47]. Antimycin A causes a collapse of the proton gradient across the inner mitochondrial membrane by blocking the mitochondrial electron transport chain, whereas oligomycin inhibits the flow of protons through F_1_F_0_ ATPase inhibition, leading to a complete Δψ_m_ collapse. Importantly, antimycin A is also a potent generator of oxidative stress, which is known to induce PINK1/Parkin-mediated mitophagy as well [48–50]. As shown by immunoblotting, antimycin A (1 μM, 24 h) alone was not sufficient to stabilize PINK1 levels in WT cells, while treatment with oligomycin (1 μM, 24 h) did (Fig. 5a, b). As seen with CCCP, the oligomycin-treated VPS35^D620N^ cells showed less accumulation of PINK1 and higher levels of Parkin compared to WT (Fig. 5a, b). Notably, co-incubation with AO caused high PINK1 accumulation and loss of Parkin in both WT and VPS35^D620N^ cells, and in a similar manner (Fig. 5a, b). To corroborate this finding, mitochondrial PINK1 accumulation was determined by IF after 24 h of 1 μM AO treatment (Fig. 5c). In agreement with our immunoblotting data, PINK1 colocalized with TOM20 in almost all WT and VPS35^D620N^ cells, and no differences in the level of colocalization were observed between the different cell lines (Fig. 5d). Finally, we monitored AO-induced mitophagy using the dual color mitophagy reporter stably expressed in WT and VPS35^D620N^ cells and observed no difference (Fig. 5e, f). Together, these findings show that PINK1/Parkin recruitment and mitophagy can occur in VPS35^D620N^ cells in response to specific kinds of mitochondrial damage. However, the type and/or severity of insult to the mitochondrial membrane potential determines whether or not PINK1/Parkin-mediated mitophagy is initiated in VPS35^D620N^ cells.

### Altered mitochondrial membrane potential and response to CCCP treatment in VPS35^D620N^ cells

To investigate whether AO treatment caused a different type of mitochondrial damage from that by CCCP treatment, we examined the rearrangement of the mitochondrial network upon exposure to these treatments. To do so, we analyzed the TOM20 distribution using IF to study the morphological characteristics of mitochondria including the number, aspect ratio and length of mitochondria in AO- and CCCP-treated cells (Fig. S2a–d). Both treatments caused mitochondrial fragmentation (Fig. S2a), as evidenced by a substantial increase in mitochondrial particles (Fig. S2b) and decreases in aspect ratio (Fig. S2c) and mitochondrial length (Fig. S2d). However, AO treatment led to more fragmentation than CCCP treatment, as the number of mitochondrial particles was significantly higher (Fig. S2a, b). In addition, consistent with our previous results, no differences were observed between WT and VPS35^D620N^ cells upon AO treatment. Interestingly, upon CCCP treatment, the mitochondrial particles appeared less rounded and longer, as reflected by the increase in aspect ratio and length, respectively, in the VPS35^D620N^ cells compared to WT cells (Fig. S2b–d). These data suggest that mitochondria in cells respond differently to AO and CCCP treatment and that AO causes more severe mitochondrial damage/fragmentation than CCCP in all cell lines. Additionally, the mitochondria in VPS35^D620N^ cells are affected by the treatments, i.e. they display mitochondrial fragmentation, albeit to a lesser extent than in WT cells.

**Fig. 6:**
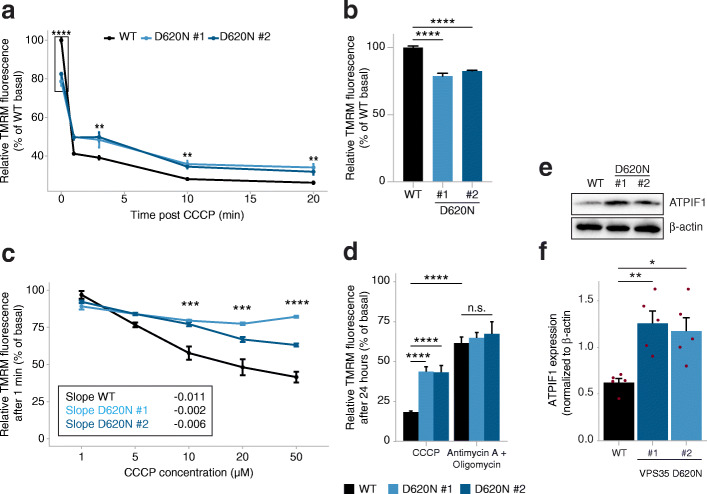
VPS35^D620N^ cells are less susceptible to CCCP-induced mitochondrial depolarization. **a** Measurements of mitochondrial membrane potential of WT and VPS35^D620N^ (clone 1 and 2) cells by TMRM fluorescence at basal level and at 1, 3, 10 and 20 min after treatment with 10 μM CCCP. **b** Basal TMRM levels from boxed area highlighted in (**a**). **c** TMRM fluorescence was measured 1 min after treatment with 1, 5, 10, 20 or 50 μM CCCP. Shown data are relative to basal conditions of each cell line. Slopes were determined using a simple linear regression. **d** TMRM fluorescence was measured 24 h after treatment with 10 μM CCCP or 1 μM antimycin A and 1 μM oligomycin. Shown data are relative to basal conditions of each cell line. Statistical analyses were performed using two-way ANOVA followed by Tukey’s *post-hoc* test. **e** Representative immunoblots of protein extracts from WT and VPS35^D620N^ (clone 1 and clone 2) cells at steady state. Blots were stained with ATPIF1 and β-actin (total protein loading control) antibodies. **f** Quantification of ATPIF1 levels from immunoblots in (**e**). Each red dot depicts a separate experiment. Statistical analyses were performed by one-way ANOVA followed by Tukey’s *post-hoc* test. *P < 0.05, **P < 0.01, ***P < 0.005, ****P < 0.001

To further explore why VPS35^D620N^ cells were affected by CCCP-induced damage but did not activate PINK1/Parkin mitophagy, we investigated the Δψ_m_ collapse upon CCCP treatment. The Δψ_m_ collapse triggers mitochondrial fragmentation, PINK1 accumulation on mitochondria and induction of mitophagy [21, 29]. Δψ_m_ was measured with the cell-permeant fluorescent dye TMRM in WT and VPS35^D620N^ cells over time upon treatment with 10 μM CCCP. Although CCCP treatment rapidly decreased Δψ_m_ in both WT and VPS35^D620N^ cells after 1 min, and the Δψ_m_ gradually decreased further during the next 19 min (Fig. 6a), the collapse in Δψ_m_ was significantly lower in VPS35^D620N^ cells compared to the WT cells at all measured time points. Additionally, the VPS35^D620N^ cells exhibited a lower Δψ_m_ at resting condition (±25% less) compared to the WT cells (Fig. 6b). To test whether the diminished Δψ_m_ reduction after 10 μM CCCP treatment in the VPS35^D620N^ cells is dose-dependent, Δψ_m_ was measured 1 min after treatment with 1, 5, 10, 20 and 50 μM CCCP. A greater Δψ_m_ reduction was observed with higher concentrations of CCCP in WT cells (slope − 0.011). In the VPS35^D620N^ cells, however, Δψ_m_ reduction remained diminished compared to the WT cells irrespective of the applied dose of CCCP (slope − 0.002 and − 0.006 for clones 1 and 2, respectively) (Fig. 6c). Finally, Δψ_m_ was measured in WT and VPS35^D620N^ cells after 24 h of treatment with 10 μM CCCP or 1 μM AO to compare the effect of these treatments. In line with the previous results, Δψ_m_ was greatly reduced after 24 h of CCCP treatment in the WT cells but diminished in the VPS35^D620N^ cells. In contrast, Δψ_m_ remained higher after 24 h of AO treatment compared to CCCP and no difference was observed between the WT and VPS35^D620N^ cells. Together, these data reveal that the mitochondrial membrane potential in the mitochondria of VPS35^D620N^ cells is already altered at steady state and could explain the altered mitochondrial susceptibility to CCCP but not to AO. We hypothesized that the difference in Δψ_m_ reduction could be partly due to a difference in levels of ATPIF1, an endogenous inhibitor of the F_1_F_0_ ATPase that affects PINK1/Parkin-mediated mitophagy [51]. ATPIF1 has been shown to maintain Δψ_m_ by blocking the reversal of the F_1_F_0_ ATPase to inhibit the outflow of protons and prevent ATP consumption [52]. Subsequently, we investigated the ATPIF1 protein levels in WT and VPS35^D620N^ cells, and found higher ATPIF1 levels in the VPS35^D620N^ cells compared to the WT cells (Fig. 6e, f), suggesting a role for ATPIF1 in the mitophagy deficits observed in VPS35^D620N^ cells.

### VPS35^D620N^ cells exhibit increased mitochondrial fragmentation and damage at steady state

**Fig. 7:**
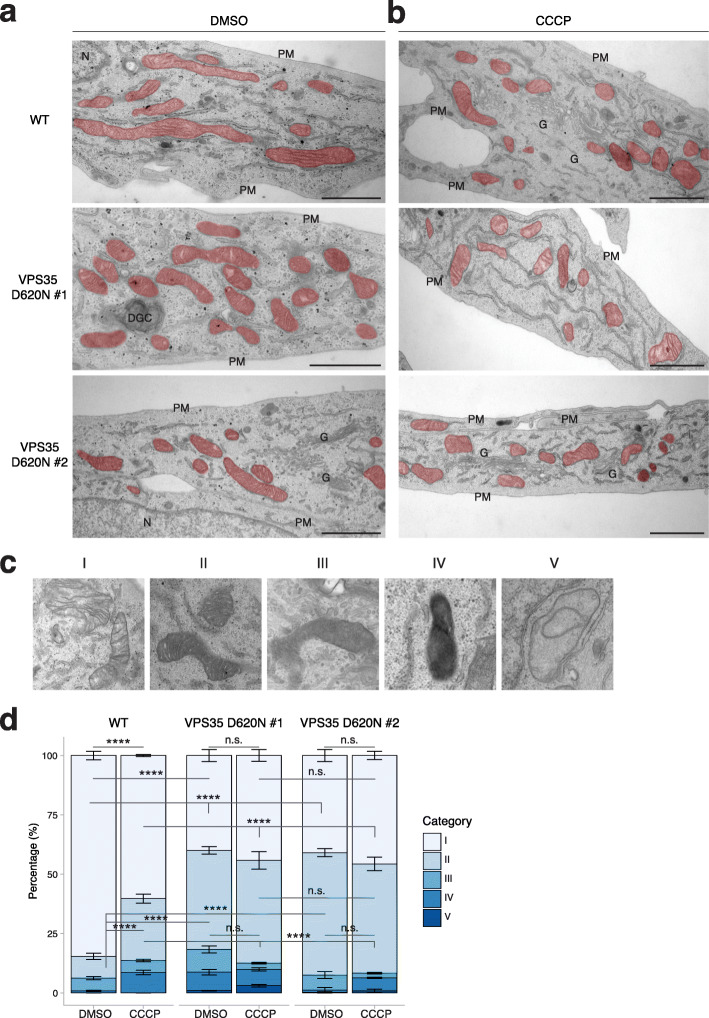
Mitochondria of VPS35^D620N^ cells appear damaged under basal conditions and do not respond to CCCP. **a, b** Representative TEM micrographs of WT and VPS35^D620N^ (clones 1 and 2) cells treated with DMSO (**a**) or 10 μM CCCP for 6 h (**b**). Mitochondria are highlighted in red. DGC, degradative compartment; G, Golgi apparatus; PM, plasma membrane. Scale bar, 1 μm. **c** Representative examples of the mitochondria categories I–V. **d** Quantification of the mitochondrial profile of WT or VPS35^D620N^ (clones 1 and 2) cells treated with DMSO or 10 μM CCCP for 6 h. Statistical analyses were performed by two-way ANOVA followed by Tukey’s *post-hoc* test. n.s. non-significant, ****P < 0.001

Given that our IF data on mitochondrial distribution were inconsistent with previous reports about cells (over)expressing VPS35^D620N^ [14, 15], likely due to the resolution limitations, we used TEM to study WT and VPS35^D620N^ cells at steady state and under CCCP-treated conditions (10 μM CCCP for 6 h). Here, we observed that the VPS35^D620N^ cells already had smaller, fragmented mitochondria compared to the WT cells at steady state (Fig. 7a), something that we had not observed with IF since the resolution of this technique is not sufficient to distinguish longer mitochondrial tubules from multiple fragmented mitochondria in close proximity. CCCP treatment led to mitochondrial fragmentation in WT cells that resembled the mitochondrial phenotype of VPS35^D620N^ cells at steady state (Fig. 7a, b). Notably, no further mitochondrial fragmentation was detected in the CCCP-treated VPS35^D620N^ cells compared to the mitochondrial fragmentation seen in the VPS35^D620N^ cells at steady state (Fig. 7b).

Furthermore, five morphologically distinct categories of mitochondria were observed in the various samples (Fig. 7c): (I) classical healthy mitochondria with well-defined cristae, (II) swollen mitochondria with defined cristae and dark in content, (III) mitochondria with unclear, partially visible cristae, (IV) mitochondria with very dark content and no visible cristae, and (V) aberrant mitochondria with remnants of cristae and light in content. At steady state, most mitochondria (~ 84%) in the WT cells were category I, and the remainder were categories II (~ 9%) and III (~ 5%). In contrast, in the VPS35^D620N^ cells at steady state, a large proportion (~ 45%) of the mitochondria were in category II, and we observed significantly fewer healthy category I mitochondria compared to the WT cells (Fig. 7d). Upon CCCP treatment, we observed a shift from category I (~ 60%) to category II (~ 26%) mitochondria in the WT cells, as well as an increase in category IV mitochondria (from ~ 1% to ~ 9%). This suggests that the category II mitochondria were damaged. Intriguingly, CCCP treatment did not cause a compositional shift in the mitochondrial population in the VPS35^D620N^ cells. These data suggest that VPS35^D620N^ cells at steady state already contain a population of damaged and fragmented mitochondria and, in agreement with our other results, confirm that this population of mitochondria does not respond further to CCCP treatment.

## Discussion

In the present study, we show for the first time that the actions of VPS35 converge on the PINK1/Parkin pathway and that the VPS35^D620N^ cells show deficits in CCCP-induced PINK1/Parkin-mediated mitophagy. Importantly, these data were acquired using a model that closely mimics the situation in PD patients. The mitochondria of VPS35^D620N^ cells seemed desensitized to a CCCP-induced Δψ_m_ collapse, as they appeared already damaged/fragmented and had a reduced mitochondrial membrane potential at steady state. Consequently, the mitochondria of CCCP-treated VPS35^D620N^ cells showed almost no accumulation of PINK1 and Parkin, and therefore failed to initiate mitophagy. However, PINK1/Parkin-dependent mitophagy in VPS35^D620N^ cells was still operational, as the VPS35^D620N^ cells displayed PINK1/Parkin-mediated mitophagy upon AO treatment. The results suggest that the mitochondria of VPS35^D620N^ already exhibit a specific type of damage at steady state. This renders them insensitive to CCCP and likely also to other stressors that may initiate PINK1/Parkin-mediated mitophagy in humans. We speculate that individuals carrying the p.D620N variant of VPS35 tend to accumulate damaged mitochondria because of this impairment, and, over time, this could cause neurodegeneration.

The observed failure of VPS35^D620N^ cells to maintain Δψ_m_ under steady state is likely linked to the presence of damaged mitochondria and will have deleterious effects on cell viability and functions, as Δψ_m_ provides the driving force for ATP synthesis [53]. Maintenance of Δψ_m_ is important for the inward transport of cations such as Ca^2+^ [54] and is necessary for the import of numerous mitochondrial proteins [55, 56]. Mitochondrial quality control mechanisms that maintain Δψ_m_, such as mitochondrial fragmentation [57] and the removal of depolarized mitochondria through mitophagy [29, 58], are thus essential and are likely affected in VPS35^D620N^ cells, which leads to the observed accumulation of damaged and fragmented mitochondria under steady state conditions. Depletion of VPS35 in neuroblastoma cells also causes reduced basal Δψ_m_ and an increase in mitochondrial fission at steady state [15]. Notably, defects in the maintenance of Δψ_m_ and mitochondrial dynamics have been observed in other models of PD, including those genetically modified for PINK1 and Parkin [59, 60]. Importantly, while this manuscript was in preparation, a study reported that patient-derived p.D620N-mutant VPS35 dopaminergic neurons exhibit a reduction in Δψ_m_ at steady state, and show a lysosomal-associated defect in CCCP-induced mitochondrial clearance [61]. While we were unable to test for mitochondrial clearance in CCCP-treated VPS35^D620N^ cells, we did not observe an evident impairment in this pathway in VPS35^D620N^ cells upon AO treatment. However, we cannot exclude that mitochondrial clearance is not affected. A mitophagy impairment downstream of mitophagy induction may therefore contribute to the accumulation of damaged mitochondria at steady state.

Our data also suggest that the VPS35^D620N^ cells are less able to respond to a collapse in Δψ_m_, as indicated by reduced PINK1/Parkin-mediated mitophagy upon Δψ_m_ loss due to CCCP treatment. The fact that the AO treatment was able to induce PINK1/Parkin-mediated mitophagy in VPS35^D620N^ cells, while CCCP could not, can be explained by the difference in how the compounds affect mitochondrial depolarization. CCCP can dissipate Δψ_m_ by removing the proton gradient over the mitochondrial membrane through increasing the permeability of protons across the inner mitochondrial membrane. As such, the duration and the amount of CCCP dictate the extent of Δψ_m_ loss, and consequently the amount of PINK1 stabilization and accumulation. Antimycin A and oligomycin both block the function of the mitochondrial electron transport chain that actively maintains the Δψ_m_, causing a loss of Δψ_m_. Oligomycin also blocks the reverse ATP synthase activity of the F_1_F_0_ ATPase, which is normally utilized to counteract the loss of Δψ_m_ by actively pumping protons into the intermembrane space, thereby causing a further decrease in Δψ_m_ [51, 62]. In addition, it is known that the inhibition of electron transport chain subcomplex III by antimycin A can cause an increase in oxidative stress through the production of reactive oxygen species [48]. Importantly and in line with our data, AO has been previously shown to cause less mitochondrial membrane depolarization compared to CCCP [50]. Therefore, the robust PINK1 accumulation in response to AO treatment is not solely due to the dissipation of Δψ_m_, which rather probably works in conjunction with increased oxidative stress to form a more substantial mitophagy stimulus than CCCP. Notably, a recent study has shown that the AO-induced PINK1 accumulation can be inhibited by antioxidants [63]. This could explain why the VPS35^D620N^ cells accumulated PINK1 when treated with AO, but not when exposed to CCCP. Furthermore, the increased ATPIF1 levels in VPS35^D620N^ cells probably contributed to the diminished Δψ_m_ dissipation in response to CCCP as the F_1_F_0_ ATPase was more inhibited. Thus, it is likely that in VPS35^D620N^ cells, which already have a lower Δψ_m_ at steady state conditions, the Δψ_m_ collapses induced by CCCP are too small to provoke additional mitochondrial fragmentation and thereby induce PINK1/Parkin-mediated mitophagy, while this is not the case with AO treatment due to the different mode of PINK1 recruitment.

So why was PINK1/Parkin-mediated mitophagy impaired in VPS35^D620N^ cells upon CCCP treatment? Since VPS35^D620N^ cells already display loss of Δψ_m_ at steady state, PINK1/Parkin-mediated mitophagy may not be activated upon mild stress, which prevents continuous turnover of mildly damaged mitochondria. As these damaged mitochondria are chronically stressed at steady state, we hypothesize that they may be desensitized by a yet unknown regulatory feedback loop or factor to prevent depletion of the mitochondrial population. Although we did not elucidate how VPS35 modifies PINK1/Parkin-mediated mitophagy, our data advocate that this impairment is associated with the already damaged/fragmented mitochondria with lower Δψ_m_ in VPS35^D620N^ cells at steady state.

Furthermore, as our study focused only on PINK1/Parkin-mediated mitophagy, we cannot rule out that other forms of mitophagy are affected in VPS35^D620N^ cells. Of note, multiple studies have shown that mitophagy can be induced with CCCP *via* other ubiquitin E3 ligases, independent of Parkin [64, 65]. Moreover, receptor-mediated mitophagy can occur without ubiquitin E3 ligases through direct interactions of autophagic receptors present on the OMM with LC3, thereby circumventing the PINK1/Parkin pathway [66, 67]. Future studies are necessary to investigate whether these alternative routes of mitophagy induction are also affected in VPS35^D620N^ cells.

One established role of VPS35 and retromer in mitochondrial physiology is to retrieve mitochondrial proteins *via *MDVs [13], and it has been shown that the D620N mutation of *VPS35* affects the sorting of MUL1 and DLP1 [14, 16]. With the expanding research on the proteome of MDVs [68], it is likely that the retromer is involved in trafficking of additional mitochondrial proteins. It is unclear to what extent this is regulated by retromer and, importantly, which cargoes are being transported. However, the sorting of other mitochondrial proteins is probably affected and could cause mitochondrial impairments, e.g. damage/fragmentation, in VPS35^D620N^ cells. For example, Δψ_m_ loss in the VPS35^D620N^ cells at steady state might be caused by changes in regulatory proteins involved in maintaining Δψ_m_, including ATPIF1, and other proteins such as the components of the mitochondrial permeability transition pore complex and the oxidative phosphorylation machinery [69, 70]. Notably, ATPIF1 has also been found enriched in MDVs [68]. Whether VPS35 plays an active role in the regulation of ATPIF1 has to be determined in future studies. In addition, a recent study has shown that VPS35 interacts with Parkin, suggesting that members of the PINK1/Parkin pathway are directly affected by VPS35-mediated MDV trafficking [71]. Although the role of VPS35 in MDV transport has been established [13, 14], the field of MDV-mediated transport is still emerging and many questions remain regarding their role in mitochondrial quality control and regulation of different cargoes [72].

## Conclusion

Our data show that the D620N variant in *VPS35* leads to mitochondrial defects that affect PINK1/Parkin-mediated mitophagy. This finding supports the notion that multiple familial PD genes converge in similar pathways and further extends our knowledge about the general disease mechanisms of PD.

## Supplementary Information

**Additional file 1: Fig. S1.** Parkin degradation is impaired in VPS35 D620N cells upon CCCP treatment. **Fig. S2.** CCCP and antimycin A and oligomycin treatments lead to rearrangements of the mitochondrial network.

**Additional file 2: Table S1.** Primers used for creating VPS35^D620N^ clones using CRISPR-Cas9. Abbreviation: *sgRNA:* single guide RNA. * indicates phosphorothioate bonds between nucleotides.

## Data Availability

The raw data and fluorescence images are available from the corresponding author upon reasonable request.

## Abbreviations

AO: Antimycin A and Oligomycin
CCCP: Carbonyl cyanide m-chlorophenyl hydrazone
IF: Immunofluorescence
PD: Parkinson’s disease
TEM: Transmission electron microscopy
TMRM: Tetramethyl-rhodamine, methyl ester
Δψ_m_: Mitochondrial membrane potential
WT: Wild type
