# Supplementary material for: Parkinson’s disease–associated VPS35 mutant reduces mitochondrial membrane potential and impairs PINK1/Parkin-mediated mitophagy
Source: Transl Neurodegener. 2021 Jun 15;10:19. doi: 10.1186/s40035-021-00243-4 (PMC8204421; doi:10.1186/s40035-021-00243-4)
Supplement: Supplementary file 1 — Additional file 1: Fig. S1. Parkin degradation is impaired in VPS35 D620N cells upon CCCP treatment. Fig. S2. CCCP and antimycin A and oligomycin treatments lead to rearrangements of the mitochondrial network. [file 40035_2021_243_MOESM1_ESM.pdf]

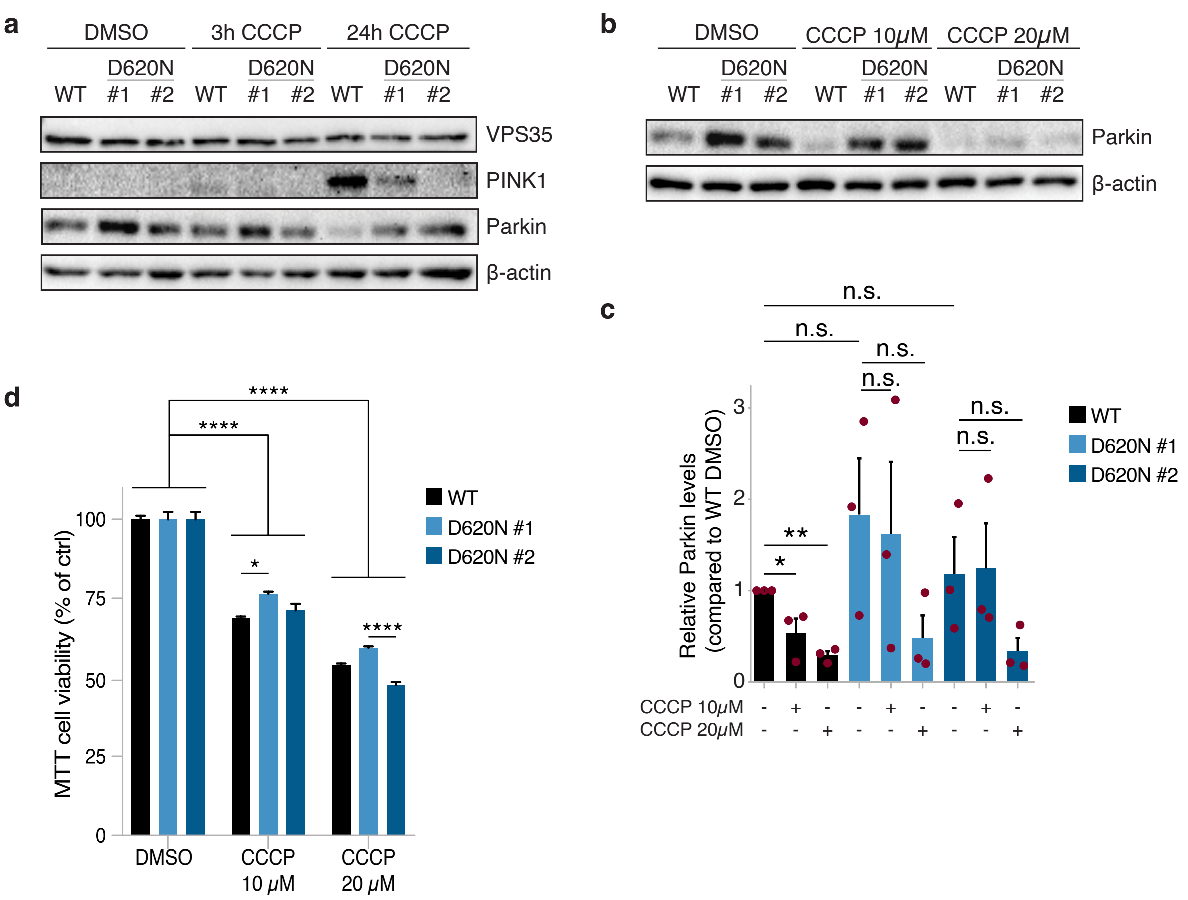

Fig. S1 Parkin degradation is impaired in VPS35 D620N cells upon CCCP treatment.

a) Representative immunoblots of protein extracts from WT and VPS35 D620N (clone 1 and clone 2) SH-SY5Y cells treated with DMSO or 10 µM CCCP for 3 or 24 h. Blots were stained for VPS35, PINK1, Parkin and β-actin (total protein loading control) antibodies. **b)** Representative immunoblots of protein extracts from WT and $VPS35^{D620N}$ (clone 1 and clone 2) cells treated with DMSO, 10 µM CCCP or 20 µM CCCP for 24 h. Blots were stained with Parkin and β-actin (total protein loading control) antibodies. **c**) Quantification of relative Parkin levels after DMSO, 10 µM CCCP or 20 µM CCCP treatment for 24 h compared to WT DMSO. Each red dot

represents a separate experiment. **d**) MTT cell viability assay of WT and VPS35^{D620N} (clone 1 and clone 2) cells upon treatment with DMSO, 10 μM CCCP or 20 μM CCCP for 24 h. Statistical analyses were performed using two-way ANOVA followed by a Tukey's *post-hoc* test. n.s. non-significant, * $p < 0.05$, ** $p < 0.01$, **** $p < 0.001$

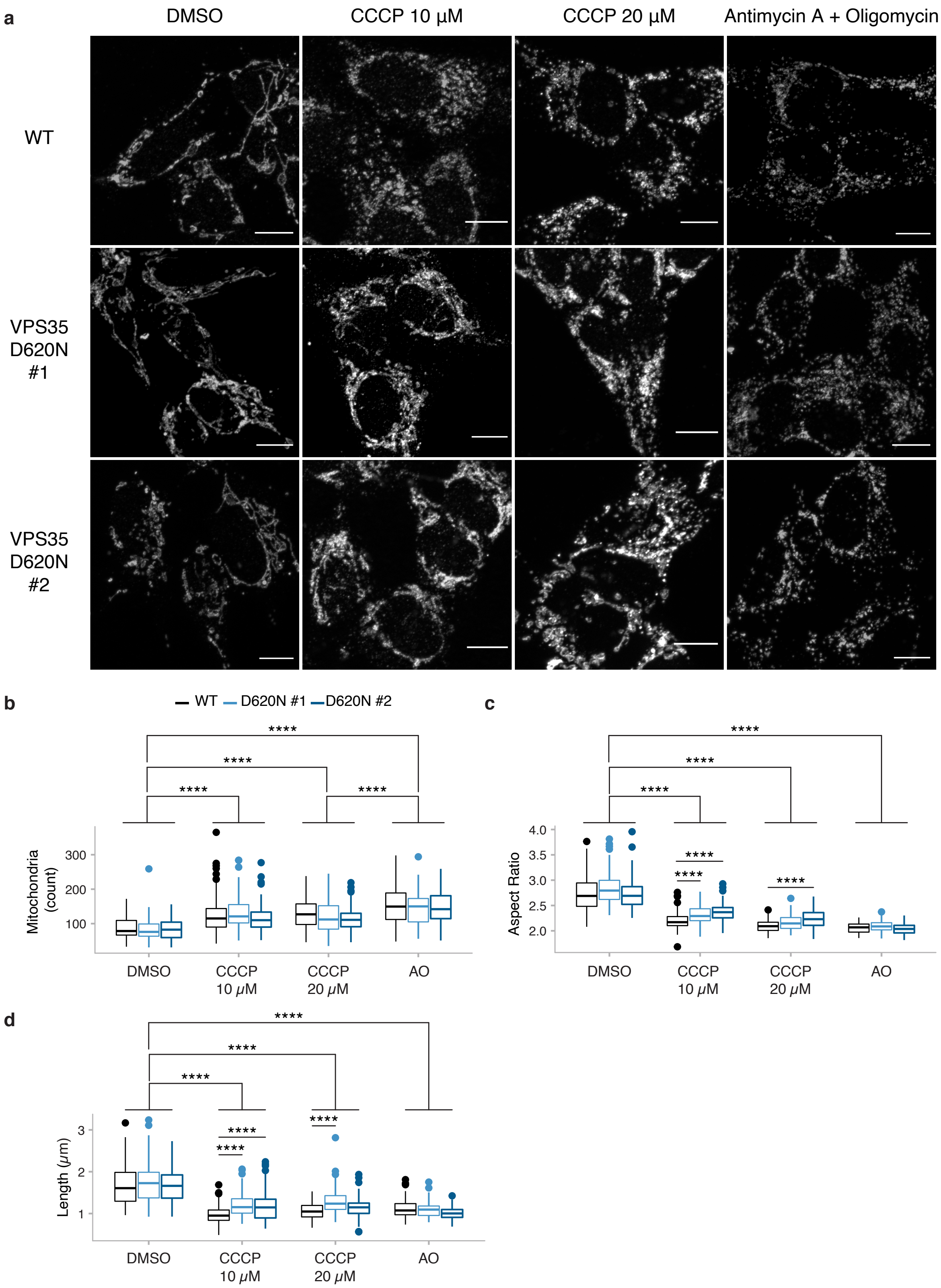

a
DMSO
CCCP 10 µM
CCCP 20 µM
Antimycin A + Oligomycin
WT
VPS35 D620N #1
VPS35 D620N #2
b
WT
D620N #1
D620N #2
****
****
****
****
Mitochondria (count)
300
200
100
DMSO
CCCP 10 µM
CCCP 20 µM
AO
c
****
****
****
****
****
****
Aspect Ratio
4.0
3.5
3.0
2.5
2.0
DMSO
CCCP 10 µM
CCCP 20 µM
AO
d
****
****
****
****
****
****
Length (µm)
3
2
1
DMSO
CCCP 10 µM
CCCP 20 µM
AO

Fig. S2 CCCP and antimycin A and oligomycin treatments lead to rearrangements of the mitochondrial network. a) Representative fluorescence images of WT or $VPS35^{D620N}$ (clone 1 and 2) cells treated with DMSO, 10 μM CCCP, 20 μM CCCP or 1 μM antimycin A and 1 μM oligomycin for 24 h stained for endogenous TOM20. Scale bar, 10 μm. **b-d)** Quantification of mitochondrial morphology in (a) represented by number of mitochondria (b), aspect ratio (c) and length (d) as determined in TOM20-stained WT and $VPS35^{D620N}$ (clone 1 and 2) cells treated with DMSO, 10 μM CCCP, 20 μM CCCP or 1 μM antimycin A and 1 μM oligomycin for 24 h. Statistical analyses were performed using two-way ANOVA followed by a Tukey's *post-hoc* test. *** $p < 0.005$, **** $p < 0.001$.
